# Disorders of the Sexual Organs in the Male

**Published:** 1896-02

**Authors:** 


### Book Reviews.

Disorders of the Sexual Organs in the Male. By Eugene Fuller, M. D., Instructor in Venereal and Genito-urinary Diseases, New York Post-Graduate Medical School. In one very handsome octavo volume of 238 pages, with twenty-five engravings and eight full-page plates. Cloth, $2.00. Philadelphia: Lea Brothers & Co., Publishers. 1895.

Dr. Fuller truly says : " It is remarkable that so little attention and scientific study should have been devoted to investigations upon this subject." He further says, "that the aim of his work is to show that pathological and physiological factors are more commonly the direct causes of sexual disorders, and that the psychological and neurotic reflexes are in the minority."

Sexual disturbances are primarily, at least, and generally, due to causes located in the sexual apparatus.

The first chapter presents a systematic study of the vesico-rectal anatomy, and the text is illustrated with seventeen original and excellent cuts.

The second chapter treats of physiology of the male sexual organs. The functions of the genital anatomy described in chapter I. are grouped under three headings : (1) The passing of the testicular secretion to the seminal vesicles ; (2) the temporary storage and production of a preservative fluid needed for the spermatozoa ; (3) the expulsion of this compound secretion as occasion requires. Dr. Fuller claims that the passing of the testicular secretion through the vas deferens into the vesicles is due largely to rhythmic action of the ampulla of Henle, and that this enlarged cavity of the vas is not a storehouse for the testicular secretion and is not in the least associated with the seminal vesicles in the act of ejaculation. Fuller contends that the ampulla and the seminal vesicles do not make one common space ; that the vas deferens connects with the vesicles by very small opening, at a sharp angle ; and through a sphincter or valve-like structure, which closes with the contraction of the vesicle during ejaculation. Further, that it would be impossible for these structures to empty themselves in unison through the ejaculatory duct ; that the valvular quality of this passage-way prevents the backward flow of this fluid into the ampulla when the vas is relaxed. Again, the ampulla is too small to cut any figure as a storehouse. The ciliated epithelium undoubtedly plays an important part in passing the testicular secretion through the two feet of vas deferens, but the entire canal is not lined with ciliated epithelium, as Dr. Ewing has shown that the ampulla is lined with columnar epithelium.

The walls of the ampulla, as shown in the chapter on anatomy, are thickened and muscular trabeculæ line the interior of this cavity. There is surely a pumping, a rhythmic action, of at least this portion of the vas deferens, which carries the testicular secretion onward into the vesicle.

This action is probably induced by the stimulus of the presence of this secretion. The function of the seminal vesicle is the storage and secretion of a preservative menstruum for the spermatozoa as well as the expulsion of the seminal fluid on call.

The vesicular epithelium and that lining the small convoluted canals, which empty into the seminal vesicle, secrete the preservative fluid and this fluid gives to the spermatic secretion its characteristics and peculiar appearance. This fluid is alkaline in reaction. Spermatozoa die immediately in acid media.

Chapter III. gives twenty-eight pages of pathology relating to morbid conditions directly affecting the male sexual function.

The pathology of the prostate is eliminated as its disturbances affect the urinary more decidedly than the sexual function. This chapter treats especially of diseases of the seminal vesicles and of peri-vesicular inflammation.

Chapter V. presents differential diagnosis. Fuller says: "If one will familiarise himself with the clinical features of seminal vesiculitis, there need be but little danger of mistaking this disease.

The diseases which it simulates are prostatitis, cystitis, posterior urethritis, stricture, peritonitis, acute appendicitis, acute epididimitis, kidney colic, and acute inflammation of the rectum.

The treatment of these maladies and many illustrative cases are given in the final chapters of this work.

Disorders of the male sexual organs is an advance upon this special work and should be in the hands of all who are called upon to do genito-urinary practice. B. H. D.

